# Mitochondrial Alterations and Enhanced Human Leukocyte/Endothelial Cell Interactions in Type 1 Diabetes

**DOI:** 10.3390/jcm9072155

**Published:** 2020-07-08

**Authors:** Francesca Iannantuoni, Aranzazu M. de Marañon, Zaida Abad-Jiménez, Francisco Canet, Pedro Díaz-Pozo, Sandra López-Domènech, Carlos Morillas, Milagros Rocha, Víctor M. Víctor

**Affiliations:** 1 Service of Endocrinology and Nutrition, University Hospital Doctor Peset, Foundation for the Promotion of Health and Biomedical Research in the Valencian Region (FISABIO), 46017 Valencia, Spain; franian@alumni.uv.es (F.I.); arantxa.mmp@gmail.com (A.M.d.M.); zaiaji@alumni.uv.es (Z.A.-J.); francisco.canet.1994@gmail.com (F.C.); pedrodpas2@gmail.com (P.D.-P.); sandra.lopez@uv.es (S.L.-D.); carlos.morillas@uv.es (C.M.); 2 CIBERehd—Department of Pharmacology and Physiology, University of Valencia, 46010 Valencia, Spain; 3 Department of Physiology, University of Valencia, 46010 Valencia, Spain

**Keywords:** type 1 diabetes, mitochondria, endothelium, inflammation, cardiovascular risk

## Abstract

Type 1 diabetes has been associated with oxidative stress. This study evaluates the rates of oxidative stress, mitochondrial function, leukocyte–endothelium interactions and adhesion molecules in type 1 diabetic patients. The study population consisted of 52 diabetic patients and 46 body-composition and age-matched controls. We assessed anthropometric and metabolic parameters, oxidative stress and mitochondrial function by evaluating reactive oxygen species (ROS) production, mitochondrial ROS production, mitochondrial membrane potential and superoxide dismutase (SOD) and catalase (CAT) expression in polymorphonuclear leukocytes from type 1 diabetic patients. In addition, we evaluated interactions between leukocytes and human umbilical vein endothelial cells (HUVEC), and serum expression of adhesion molecules (P-selectin, VCAM-1 and ICAM-1), proinflammatory cytokines (IL-6 and TNFα) and myeloperoxidase (MPO). HbA_1C_ and glucose levels were higher in diabetic patients than in control subjects, as expected. Mitochondrial function was altered and leukocyte–endothelium interactions were enhanced in diabetic patients, which was evident in the increase in total and mitochondrial ROS production, higher mitochondrial membrane potential, enhanced leukocyte rolling and adhesion, and decreased rolling velocity. Furthermore, we observed an increase in levels of adhesion molecules P-selectin, VCAM-1, and ICAM-1 in these subjects. In addition, type 1 diabetic patients exhibited an increase in proinflammatory mediators TNFα and MPO, and a decreased expression of SOD. The enhancement of leukocyte–endothelium interactions and proinflammatory markers correlated with glucose and HbA_1C_levels. Mitochondrial alteration, oxidative stress, and enhanced leukocyte–endothelium interactions are features of type 1 diabetes and may be related to cardiovascular implications.

## 1. Introduction

Mitochondrial dysfunction and cardiovascular events have been previously related to metabolic diseases such as obesity and diabetes. Furthermore, impaired mitochondrial energetics and oxidative damage have been implicated in cardiac dysfunction in obesity and diabetes [1,2,3], and a disruption of physiological reactive oxygen species (ROS) homeostasis may lead to the impairment of β-cell function [4]. On the other hand, a regulated production of ROS can be beneficial, as it mediates different physiological processes [5], acting as a double-edged sword by modulating insulin signaling-required for insulin to exert its physiological action-while also playing a role in the pathogenesis of insulin resistance [3].

Cardiovascular disease (CVDs) and related macro- and microvascular disease are long-term complications of obesity and diabetes and, together with chronic diabetic renal disease, are the most important cause of morbi-mortality in patients with type 1 diabetes [6,7,8,9]. It has been estimated that the relative risk of CVDs in these patients is 10 times higher than in non-diabetics [7]. In this sense, it is important to highlight that one of the key factors in the development of CVDs is atherosclerosis. The atherosclerotic process is considered a chronic inflammatory disease characterized by accumulation of lipids, infiltration of leukocytes, proliferation of smooth muscle cells and deposition of connective tissue [10]. Furthermore, atherosclerotic lesions in type 1 diabetic patients are characterized by the involvement of multiple vessels, including the distal coronary artery, and severe stenosis [11]. The complex relationship between type 1 diabetes and CVDs is not fully understood. In fact, the longer duration of type 1 diabetes can promote CVDs through different pathophysiological pathways [7,12,13].

Peripheral polymorphonuclear leukocytes (PMNs) are one of the main types of inflammatory cells and can release ROS when activated, contributing to oxidative stress, inflammation and endothelial impairment. Eventually, oxidative stress is produced in the PMNs of insulin-resistant patients and is related to an impairment of mitochondrial function [14]. In agreement with this idea, PMNs could also be activated by glucose homeostasis variation, such as acute hyper-and hypoglycemia, and these variations can concur, generating advanced glycosylation-end products (AGEs) that promote cellular dysfunction and endothelial expression of several adhesion molecules, such as vascular adhesion molecule-1 (VCAM-1), intercellular adhesion molecule-1 (ICAM-1) and selectins [15,16,17]. On the other hand, AGEs have been demonstrated in atherosclerotic lesions of diabetic patients [18]. Furthermore, it is important to stress that the onset of many CVDs is heralded by the movement and accumulation of leukocytes in the vessel wall [18], and that these processes are mediated by an interaction between the adhesion molecules expressed on leukocytes and/or endothelial cells [19].

Lastly, PMNs also play an important role by releasing high amounts of ROS from different sources, such as mitochondria and myeloperoxidase (MPO) [20,21,22], which are well known risk factors of CVDs. Furthermore, the association of oxidative stress and inflammation with type 1 diabetes is partially documented, while the role of oxidative stress in PMNs, leukocyte–endothelium interactions and endothelial dysfunction is still unclear. The purpose of the present study was to throw light on the relationship between mitochondrial dysfunction, leukocyte activation and, especially, the increase of leukocyte–endothelium interactions observed in type 1 diabetes.

## 2. Experimental Section

### 2.1. Patients and Sample Collection

Our study population was composed of 52 patients with type 1 diabetes diagnosis [23] and 46 healthy volunteers recruited from the Endocrinology and Nutrition Service at University Hospital Dr Peset (Valencia, Spain), and who were adjusted by sex and age. The subjects signed a written informed consent form and protocols were approved by our hospital’s Ethics Committee for Clinical Investigation (ID: 98/19), in line with the ethical principles of the Helsinki declaration.

Exclusion criteria were: presence of any documented history of CVDs (ischemic cardiopathy, stroke, peripheral arteriopathy, or any other chronic disease related to cardiovascular risk); any severe condition including inflammatory, infectious, and autoimmune diseases or malignancies.

### 2.2. Clinical and Biochemical Analysis

All subjects recruited for the present study underwent a physical examination to obtain anthropometrical measurements such as weight (kg), height (m), waist circumference (cm), body mass index (BMI), and systolic (SBP) and diastolic blood pressure (DBP). Blood samples for biochemical and experimental procedures were collected from the antecubital vein between 8:00 and 9:30 a.m. in a fasting condition. To obtain serum, samples were centrifuged at 1.500 g at 4 °C, for 10 min. Glucose levels were determined by means of an automated enzymatic technique using a Beckman Synchron LX20 Pro analyzer (Beckman Coulter, Brea, CA, USA), and percentage of glycated hemoglobin A_1C_ (HbA_1C_) was obtained with an automatic glycohemoglobin analyzer (Arkray Inc., Kyoto, Japan). Total cholesterol, high-density lipoprotein cholesterol (HDL-c) and triglyceride levels were determined by means of an automated enzymatic technique (Beckman Coulter, Brea, CA, USA). Low-density lipoprotein cholesterol (LDL-c) was calculated with Friedewald’s formula. Highly sensitive C-reactive protein (hs-CRP) concentration was calculated by latex-enhanced immunonephelometric assay (Behring Nephelometer II; Dade Behring, Inc., Newark, NJ, USA).

### 2.3. Isolation of Leukocytes

To isolate human leukocytes, blood collected in citrated tubes was incubated for 45 min with dextran (3% *w/v* in saline solution; Sigma Aldrich, St. Louis, MO, USA). A gradient centrifugation was performed by placing collected supernatants over Ficoll–Hypaque (GE Healthcare, Barcelona, Spain) and centrifuging at 650 g for 25 min at room temperature. Pellets containing PMNs were collected and washed with phosphate-buffered saline (PBS; Sigma Aldrich, St. Louis, MO, USA), resuspended in 1 mL of Hank’s Balanced Salt Solution (HBSS; Sigma Aldrich, St. Louis, MO, USA). Cells were counted and divided into different aliquots: a first aliquot of 1.95 × 10^6^ cells/mL was resuspended in HBSS to perform a fluorescent microscopy assay; a second aliquot of 1.2 × 10^6^ cells/mL was resuspended in complete Roswell Park Memorial Institute medium (RPMI; Biowest-bw, Nuaillé, France) to perform leukocyte–endothelium interaction assays; the remaining cells were stored at −80 °C.

### 2.4. ROS Production and Mitochondrial Membrane Potential

Total and mitochondrial ROS production was evaluated by static fluorimetry using a fluorescence microscope (IX81; Olympus, Hamburg, Germany) coupled with the static cytometry software “ScanR” (Olympus, Hamburg, Germany). After extraction, 1.5 × 10^5^ of fresh isolated PMNs were seeded per well in 48-well plates and incubated with 2′,7′-dichlorodihydrofluorescein diacetate (DCFH-DA, 5 μM), red mitochondrial superoxide indicator (MitoSOX, 5 μM) and tetramethylrhodaminemethylester (TMRM, 5 μM) fluorescent probes (Thermo Fisher Scientific, Needham, MA, USA) for 30 min to measure total and mitochondrial ROS production, and mitochondrial membrane potential, respectively. Hoechst 33,342 (Sigma Aldrich, St Louis, MO, USA) was used to visualize nuclei. Results were expressed as % of the control.

### 2.5. Interaction and Adhesion Assays

Interaction and adhesion dynamics were explored using an ex vivo model which simulates human physiological conditions, with an inverted microscope (Nikon Eclipse TE 2000-S; Amstleveen, Netherlands) connected to a video camera (Sony Exware HAD; Koln, Germany) for visualization and a parallel plate flow-chamber system as described previously [24]. To recreate the human endothelium, human umbilical vein endothelial cells (HUVEC) were extracted from umbilical cords donated by healthy volunteers. HUVEC primary cultures were cultured in EBM2 medium (PromoCell GmbH, Heidelberg, Germany) supplemented with Supplement Pack Endothelial Cell GM2 (PromoCell GmbH, Heidelberg, Germany) on fibronectin-coated plastic coverslips (Sigma Aldrich, St. Louis, MO, USA) until confluence. To perform the assay, coverslips were placed on the bottom of the flow chamber and the leukocyte suspension—Obtained as described previously—Were perfused across the system at a flow rate of 0.36 mL/min (physiological human rates) for 5 min. The whole experiment was recorded and leukocyte rolling flux, rolling velocity and adhesion were studied as usual [24].

### 2.6. Adhesion Molecule and Biomarkers of Inflammation

To better understand cell adhesion dynamics, levels of P-selectin, VCAM-1, ICAM-1 and selected mediators of inflammation interleukin-6 (IL-6), tumor necrosis factor alpha (TNFα) and MPO were evaluated in the serum of participants using a Luminex 200 flow analyzer system (Millipore, Austin, TX, USA).

### 2.7. Quantitative Analysis of SOD and CAT Gene Expression

Total RNA from leukocyte pellets were isolated with GeneAll^®^ RibospinTM kit (GeneAll Biotechnology, Hilden, Germany). RNA concentration was measured with Nanodrop 2000 c (Life Technologies, Thermo Fisher Scientific, Needham, MA, USA), and an optical density absorption ratio of 260/280 nm between 1.8 and 2.0 confirmed RNA purity.

First-strand cDNA was attained by means of Revert Aid First-Strand cDNA Synthesis kit (Life Technologies, Thermo Fisher Scientific, Needham, MA, USA) using oligo (DT) primers. We made diluted 1:10 (*v*/*v*) working aliquots of the first-strand cDNA and 2µl of these aliquots were used in the following steps. qRT-PCR reactions were carried out as follows: 10 min at 95 °C, 40 cycles (designed in one step) at 95 °C for 10 s and one cycle at 60 °C for 30 s, and a melting curve stage using LightCycler^®^ 480 SYBR Green I Master (Roche, Mannheim, Germany) in a 7500 Fast real-time PCR system (Life Technologies, Carlsbad, CA, US).

Primers used for the study of mRNA expression of SOD1 were: Forward, GGTGTGGCCGATGTGTCTAT and Reverse, TTCCACCTTTGCCCAAGTCA; and for CAT were: Forward, CTTCGACCCAAGCAACATGC and Reverse, CGGTGAGTGTCAGGATAGGC. Data were normalized to β-Actin by using the primers: Forward, CCTCGCCTTTGCCGATCC and Reverse, CGCGGCGATATCATCATCC. Relative quantification analysis was made according the comparative 2^−ΔΔCt^ method with Expression Suite software (Life Technologies, Carlsbad, CA, USA).

### 2.8. Statistical Analysis

GraphPad Prism version 7.00 (GraphPad Software, La Jolla, CA, USA) was employed to perform the statistical analysis. Parametric variables are expressed as mean ± standard deviation (SD) and non-parametric variables as median (25th and 75th percentiles). The bar graphs in the figure show mean ± standard error of the mean (SEM). An unpaired Student’s t-test was used for comparisons between groups. Correlations between variables were explored using SPSS 17.0 software (SPSS Statistics, Inc. Chicago, IL, USA) and calculated using Spearman’s correlation coefficient. Differences were considered significant when *p* < 0.05.

## 3. Results

### 3.1. Characteristics of the Study Population

The present study involved 52 type 1 diabetic subjects and 46 healthy controls. Evolution time of the patients’ diabetes was 15.8 ± 8.3 years. Table 1 shows non-statistical differences among the groups with respect to sex, age and BMI. Type 1 diabetic patients received continuous antidiabetic treatment consisted of fast-acting insulin (Humalog, Novorapid or Fiasp), and slow-acting insulin (Tresiba, or Toujeo), and as shown in Table 1, antihypertensive medications and lipid-lowering drugs. No subjects were being treated with diuretic agents. The following comorbidities were reported among the type 1 diabetic group: 15 patients were affected by retinopathy, 3 by nephropathy and 3 by neuropathy.

Anthropometric and metabolic characteristics of the study population are shown in Table 1. We observed significant differences in glucose and HbA_1C_ levels according to diabetes definition (both *p* < 0.001). A reduction in total and LDL-c levels (both *p* < 0.05) was also observed in type 1 diabetic group, probably due to the lipid-lowering treatment received by patients. No other significant differences were found.

### 3.2. ROS Production and Membrane Potential

**Figure 1:**
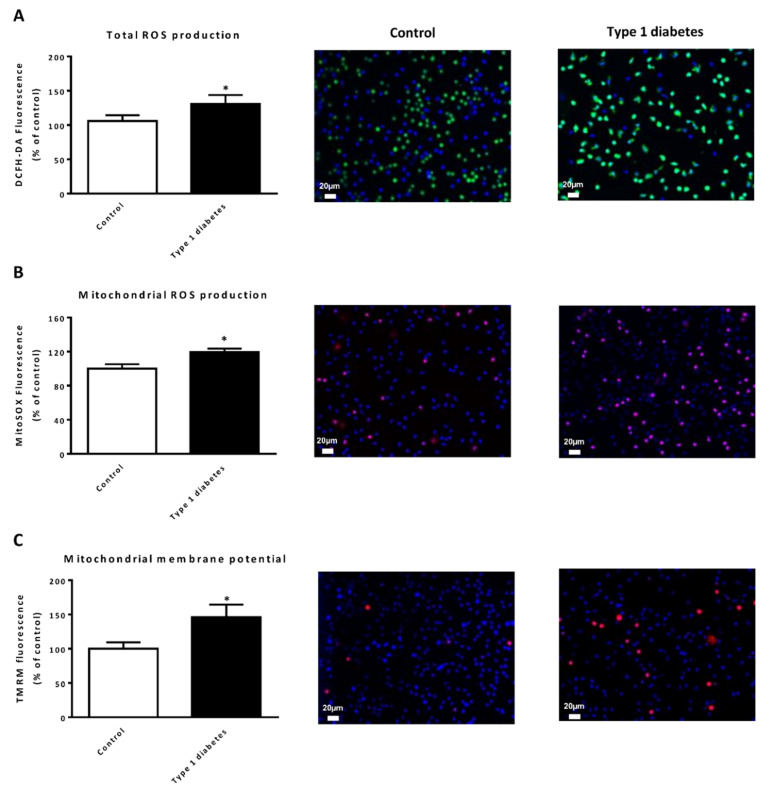
Evaluation of oxidative stress (total and mitochondrial ROS production) and mitochondrial membrane potential in type 1 diabetic and control subjects. (**A**) Levels of total ROS production measured as DCFH-DA fluorescence. Representative images of leukocytes stained with DCFH-DA (green) and Hoechst 33,342 (blue) and visualized by fluorescence microscopy. (**B**) Levels of mitochondrial ROS production measured as MitoSOX red fluorescence. Representative images of leukocytes stained with MitoSOX (red) and Hoechst 33,342 (blue) by fluorescence microscopy. (**C**) Mitochondrial membrane potential measured as TMRM fluorescence. Representative images of leukocytes stained with TMRM (red) and Hoechst 33,342 (blue) and visualized by fluorescence microscopy. Fluorescence was measured as arbitrary units of fluorescence and expressed as % of control fluorescence. Data are expressed as mean ± SEM. * *p* < 0.05 control vs. type 1 diabetic subjects.

When we focused on ROS production in leukocytes, we noticed that both total and mitochondrial ROS levels were higher in type 1 diabetic patients than in control subjects (Figure 1A,B, *p* < 0.05 both), and an increase in mitochondrial membrane potential was observed in the former group (Figure 1C, *p* < 0.05).

### 3.3. Leukocyte–Endothelium Cell Interaction

**Figure 2:**
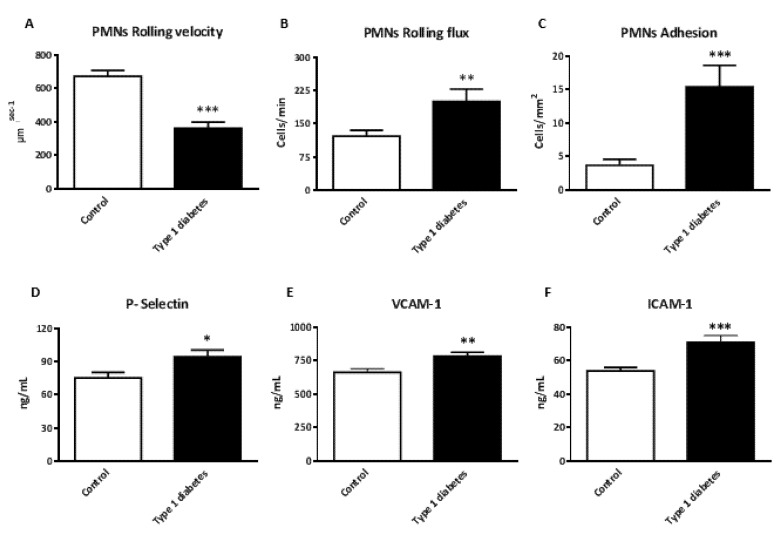
Evaluation of PMNs interactions in type 1 diabetic and control subjects, and levels of adhesion molecules in serum. (**A**) PMNs rolling velocity (μmsecond^−1^); (**B**) PMN rolling flux (cells/min); (**C**) PMNs adhesion (cells/mm^2^); (**D**) Levels of P-selectin (ng/mL); (**E**) Levels of VCAM-1 (ng/mL); (**F**) Levels of ICAM-1 (ng/mL). Data are expressed as mean ± SEM. * *p* < 0.05, ** *p* < 0.01, *** *p* < 0.001 control vs. type 1 diabetic subjects. Abbreviations: ICAM-1: intercellular adhesion molecule-1; PMNs, polymorphonuclear leukocytes; P-selectin: platelet selectin; VCAM-1: vascular cell adhesion molecule-1.

The dynamic between isolated PMNs and endothelial cells was explored using a flow-chamber system that measures the flux of PMNs, their velocity and their adhesion to the endothelium. In addition, we chose to evaluate some representative soluble adhesion molecules in serum. The results are shown in Figure 2. PMNs from type 1 diabetic patients showed a decrease in rolling velocity (Figure 2A, *p* < 0.001) and an increase in rolling flux (Figure 2B, *p* < 0.01) compared with those from controls. In addition, we observed an increase in PMNs adhesion (Figure 2C, *p* < 0.001). In line with these results, we detected an increase in the levels of the adhesion molecules P-selectin (Figure 2D, *p* < 0.05), VCAM-1 (Figure 2E, *p* < 0.01) and ICAM-1 (Figure 2F, *p* < 0.001) in type 1 diabetic patients.

### 3.4. Expression of Inflammatory Mediators

**Figure 3:**
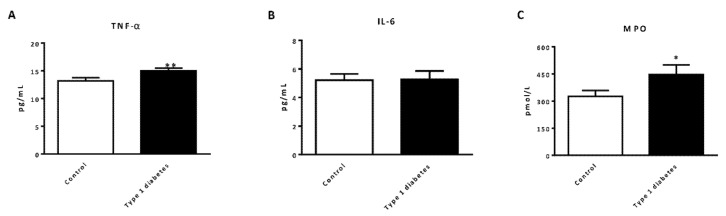
Evaluation of serum levels of cytokines and mediators of inflammation in type 1 diabetic and control subjects. (**A**) Serum levels of TNFα (pg/mL); (**B**) Serum levels of IL-6 (pg/mL); (**C**) Serum levels of MPO (pmol/L). Data are expressed as mean ± SEM. * *p* < 0.05, ** *p* < 0.01 control vs. type 1 diabetic subjects. Abbreviations: IL-6, interleukin 6; MPO, myeloperoxidase; TNFα: tumor necrosis factor alpha.

As expected, when serum TNFα levels were analyzed the data revealed an increase in type 1 diabetic patients with respect to controls (Figure 3A, *p* < 0.01), but differences in IL-6 serum levels were not found between the two groups (Figure 3B). Lastly, when we explored MPO levels, we observed an increase in serum levels in type 1 diabetic patients versus controls (Figure 3C, *p* < 0.01).

### 3.5. Antioxidant Gene Expression

**Figure 4:**
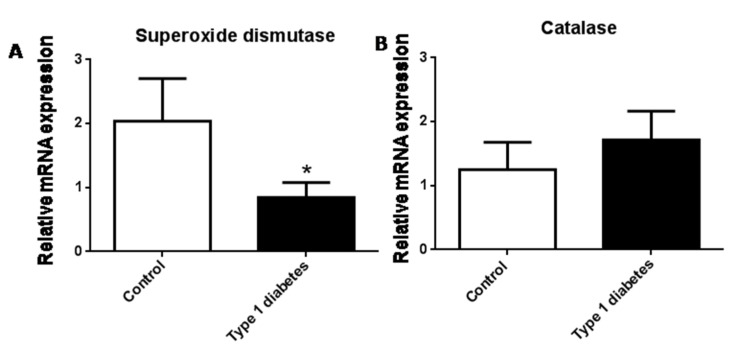
Evaluation of mRNA expression of SOD (**A**) and CAT (**B**) in type 1 diabetic and control subjects. Data are expressed as mean ± SEM. * *p* < 0.05, control vs. type 1 diabetic subjects. * *p* < 0.05 control vs. type 1 diabetic subjects.

We have measured the antioxidant gene expression of SOD and CAT. Figure 4A shows that there was a decrease in type 1 diabetic patients with respect to controls (Figure 4A, *p* < 0.05), but differences in CAT levels were not found between the two groups (Figure 4B).

### 3.6. Correlations Studies

We further explored possible correlations with glucose and HbA_1C_ levels in the study population (Table 2). Glucose was negatively correlated with rolling velocity (r = −0.457; *p* < 0.05) and positively correlated with cellular adhesion (r = 0.369; *p* < 0.001), to ICAM-1 (r = 0.335; *p* < 0.05) and VCAM-1 (r = 0.291; *p* < 0.05). In addition, HbA_1C_ was negatively correlated with rolling velocity (r = −0.719; *p* < 0.001), and positively correlated with rolling flux (r = 0.401; *p* < 0.05), cellular adhesion (r = 0.658; *p* < 0.001), ICAM-1 (r = −0.497; *p* < 0.001), VCAM-1 (r = 0.397; *p* < 0.01) and TNFα (r = 0.397; *p* < 0.05). TNFα was positively correlated with mitochondrial membrane potential (r = −0.406; *p* < 0.05), rolling flux (r = 0.469; *p* < 0.05) and MPO (r = 0.402; *p* < 0.05). Total ROS production was negatively correlated with rolling velocity (r = −0.395; *p* < 0.05), and VCAM-1 was correlated with IL-6 (r = 0.491; *p* < 0.01), while MPO correlated with hs-CRP (r = 0.427; *p* < 0.05). It is important to take into account that r < 0.5 has been considered to be a week correlation.

## 4. Discussion

The results of the present study show an increase in fasting levels of HbA_1C_ and glucose in type 1 diabetic patients with respect to controls, as expected. Several animal and human studies have provided evidence that hyperglycemia can cause pathological changes in endothelial cells through different pathways, such as activation of protein kinase C, AGEs and polyol and hexosamine pathways [13,25,26,27]. In addition to these alterations, the present study highlights mitochondrial alterations (defined as an increase in total and mitochondrial ROS production and mitochondrial membrane potential) in PMNs from type 1 diabetic patients. Mitochondria are one of the most important sources of ROS production, and play a key role in diabetes [28,29]. Physiologically, ROS are released by inflammatory cells and the endothelium, and an exacerbated ROS production can damage cells and lead to mitochondrial dysfunction and oxidative stress in β-cells, which are related to type 1 diabetes [18,27]. For these reasons, restoring mitochondrial ROS homeostasis could be essential for preventing diabetic complications and improving endothelial function in CVDs, as demonstrated by several meta-analysis studies on antioxidant supplementation in diabetic patients [30,31,32]. Furthermore, we should remember that type 1 diabetes is an autoimmune disease; it has been shown that mitochondrial function is essential for T cell activation and function and antigen presentation (as naive and memory cells depend on efficient mitochondrial oxidative phosphorylation) [33,34,35].

We have observed an increase in mitochondrial membrane potential in leukocytes from type 1 diabetic patients. In line with this, Jing et al., described an inner-membrane hyperpolarization of mitochondria in human T cells from type 1 diabetics subjects that did not correlate with metabolic control, subject age or duration of diabetes, but was correlated with ROS levels and subsequent IFNγ production [35]. In accordance with our results, it has been described that β cells exposed to hyperglycemia are prone to high levels of reducing equivalents and consumption of adenosine diphosphate (ADP) [36], resulting in an enhanced mitochondrial membrane potential and, therefore, enhanced ROS production.

The role of mitochondrial dysfunction in leukocytes has been related to vascular complications such as silent myocardial ischemia [37], but this has not been explored in depth in type 1 diabetes. To take a step further and investigate these dynamics in type 1 diabetes, we have developed an ex vivo model in which human leukocytes flow over a monolayer of human endothelial cells with shear stress [37]. This methodology mimics the process that occurs during the atherosclerosis in vivo (rolling and adhesion), which is critical for vascular integrity and homeostasis. In the present study, we have observed that type 1 diabetic patients display increased leukocyte–endothelium interactions; specifically, we have observed decreased PMN rolling velocity and increased rolling flux and adhesion, which are results of great relevance. It is important to highlight that our system has been widely used for analyzing the recruitment of leukocytes to the endothelium [38], and that this process has been poorly studied in type 1 diabetes until now. To visualize the whole adhesion cascade, we explored some of the adhesion molecules and inflammatory mediators involved in this process. We observed an increase of the adhesion molecules P-selectin, VCAM-1 and ICAM-1 expression, together with proinflammatory markers TNFα and MPO. Hypertension, CVDs and the atherosclerotic process are characterized by leukocyte recruitment to the vascular wall. In fact, when leukocyte–endothelium interactions are exacerbated, vascular dysfunction arises, leading to CVDs [39]. In addition, we have demonstrated a decrease of SOD expression, highlighting the oxidative stress situation under type 1 diabetes condition.

Lastly, we demonstrate that levels of glucose are negatively correlated with rolling velocity and positively correlated with cellular adhesion, ICAM-1 and VCAM-1, thus supporting a role for glycemic control in inflammation and the atherosclerotic process [18], as illustrated previously in animal models [40]. In support of this hypothesis, HbA_1C_ levels were also negatively correlated with rolling velocity and positively correlated with rolling flux, cellular adhesion, ICAM-1, VCAM-1 and TNFα. In this way, we can affirm that hyperglycemia enhances levels of HbA_1C_ and promotes mitochondrial impairment, leading to enhanced leukocyte–endothelium interactions [36]. In this sense, increased leukocyte–endothelium interaction has been associated with insulin resistance [24,29]. In line with the present study, a decreased venular shear rate and an enhanced number of rolling leukocytes in mesenteric vessels have previously been reported in diabetic patients with respect to controls in study employing an animal model [41].

Adhesion molecules such as P-selectin, VCAM-1 and ICAM-1 are key factors resulting from endothelial activation. In the present study, we have shown that type 1 diabetic patients exhibit an increase in these adhesion molecules. Furthermore, our data for leukocyte–endothelium interactions demonstrate an enhanced production of vascular adhesion molecules, thus providing strong evidence of the inflammatory process that occurs in type 1 diabetes and which can eventually lead to CVDs [18]. In addition, we report an increase of TNFα, a trend related to an impairment in insulin signaling [42]. Furthermore, different studies have shown that increased levels of MPO, IL-6 and hs-CRP are significantly associated with an increased risk of diabetes [22,43]. Our data show a rise in MPO levels and a correlation with hs-CRP levels, prompting the hypothesis that this enzyme is also important in the development of microvascular alterations in type 1 diabetes [44].

## 5. Conclusions

In conclusion, the current study provides evidence of oxidative stress, mitochondrial alterations, antioxidant decrease and especially enhanced leukocyte–endothelium interactions in leukocytes of type 1 diabetic patients. It is important to highlight the role of increased leukocyte–endothelium interactions and their possible role in the development of atherosclerosis in type 1 diabetes. However, knowledge of the role of mitochondrial alterations, ROS and leukocyte–endothelium interactions in type 1 diabetes is still insufficient. Future studies should focus on these features to clarify the nature of the molecular mechanisms involved in type 1 diabetes and to modulate them in both type 1 diabetes and related CVDs.

## Figures and Tables

**Table 1:** Anthropometric characteristics, biochemical determination and pharmacological treatment of the study population.

Characteristics	Control	Type 1 Diabetic Patients	*p*-Value
*n*	46	52	
Sex (% men)	46%	50%	
Age (years)	43.4 ± 7.9	41.7 ± 13.0	ns
Duration of diabetes (years)	-	15.8 ± 8.3	
Weight (kg)	73.3 ± 16.0	75.2 ± 17.0	ns
Waist circumference (cm)	84.6 ± 13.1	87.1 ± 13.4	ns
BMI (Kg/m^2^)	25.2 ± 4.7	25.3 ± 3.6	ns
SBP (mmHg)	117.8 ± 13.3	120.0 ± 19.6	ns
DBP (mmHg)	70.2 ± 8.4	71.4 ± 8.2	ns
Glucose (mg/dL)	91.9 ± 11.3	167.9 ± 74.8***	*p* < 0.001
HbA_1C_-DCCT (%)	5.21 ± 0.22	7.72 ± 1.12***	*p* < 0.001
Total cholesterol (mg/dL)	189.9 ± 32.7	171.5 ± 29.2	ns
HDL-c (mg/dL)	58.2 ± 12.4	57.8 ± 14.6	ns
LDL-c (mg/dL)	115.1 ± 26.8	98.6 ± 26.4	ns
Triglycerides (mg/dL)	73.0 (54.0–104.0)	73 (53.0–87.0)	ns
hs-CRP (mg/L)	1.15 (0.48–2.0)	1.17(0.56–2.32)	ns
**TREATMENT (%)**			
Antihypertensive medications	-	9.6%	
Statins	-	40.4%	
Fibrate	-	3.9%	

Data are expressed as mean ± SD for parametric variables and as median (interquartile range) for non-parametric data. An unpaired Student’s t-test was used for comparisons between groups.*** *p* < 0.001 control vs. type 1 diabetic subjects. Abbreviations: BMI, body mass index; DBP, diastolic blood pressure; HbA_1C_-DCCT, glycated hemoglobin-according to Diabetes Control and Complications Trial method; HDL-c, high-density lipoprotein cholesterol; HOMA-IR, homeostasis model assessment of insulin resistance; hs-CRP, high-sensitive C-reactive protein; LDL-c, low-density lipoprotein cholesterol; ns, not significant; SBP, systolic blood pressure.

**Table 2:** Correlations between glucose blood, ROS production, leukocyte–endothelium interaction and inflammation in study population.

Parameters	Glucose		HbA_1C_-DCCT		Total ROS Production		TNFα		hs-CRP	
**Rolling velocity**	r = −0.457	*p* < 0.05	r = −0.719	*p* < 0.001	r = −0.395	*p* < 0.05	-		-	
**Rolling flux**	-		r = 0.401	*p* < 0.05	-		r = 0.469	*p* < 0.05	-	
**Cellular adhesion**	r = 0.369;	*p* < 0.001	r = 0.658	-	-		-		-	
**ICAM-1**	r = 0.335	*p* < 0.05	r = 0.497	-	-		-		-	
**VCAM-1**	r = 0.291	*p* < 0.05	r = 0.397	-	-		-		-	
**TNFα**	-		r = 0.397	*p* < 0.05	-		-		-	
**IL-6**	-		-		-		-		-	
**MPO**	-		-		-		r = 0.402	*p* < 0.05	r = 0.427	*p* < 0.05
**Membrane potential**	-		-		-		r = 0.406	*p* < 0.05	-	

Correlation coefficients were estimated by Pearson’s correlation for parametric parameters or Spearman’s correlation when data were nor normally distributed. Abbreviations: HbA_1C_-DCCT, glycated hemoglobin HbA_1C_-according to Diabetes Control and Complications Trial method; hs-CRP, highly sensitive C-reactive protein; ICAM-1, intercellular adhesion molecule-1; IL-6, interleukin-6; MPO, myeloperoxidase; ROS, reactive oxygen species TNF-α, Tumor necrosis factor alpha; VCAM-1, vascular cell adhesion molecule-1.
